# Metformin: When Should We Fear Lactic Acidosis?

**DOI:** 10.3390/ijms23158320

**Published:** 2022-07-28

**Authors:** Stefania Di Mauro, Agnese Filippello, Alessandra Scamporrino, Francesco Purrello, Salvatore Piro, Roberta Malaguarnera

**Affiliations:** 1 Department of Clinical and Experimental Medicine, Internal Medicine, Garibaldi-Nesima Hospital, University of Catania, 95122 Catania, Italy; 8stefaniadimauro6@gmail.com (S.D.M.); agnese.filippello@gmail.com (A.F.); alessandraska@hotmail.com (A.S.); fpurrell@unict.it (F.P.); 2 Faculty of Medicine and Surgery, “Kore” University of Enna, 94100 Enna, Italy; roberta.malaguarnera@unikore.it

**Keywords:** metformin, biguanides, MALA (metformin-associated lactic acidosis)

## Abstract

Metformin, a molecule belonging to the biguanide family, represents one of the most commonly prescribed medications for the treatment of diabetes mellitus in the world. Over the sixty years during which it has been used, many benefits have been described, which are not limited to the treatment of diabetes mellitus. However, since metformin is similar to other members of the same drug family, there is still much concern regarding the risk of lactic acidosis. This article aims to highlight the correlation between the use of metformin and the onset of renal damage or lactic acidosis. Metformin-associated lactic acidosis exists; however, it is rare. The appropriate use of the drug, under safe conditions, induces benefits without risks.

## 1. Introduction

Metformin, a molecule belonging to the biguanide family, is among the most well-known and most commonly used drugs for the treatment of diabetes mellitus in the world.

**Figure 1:**
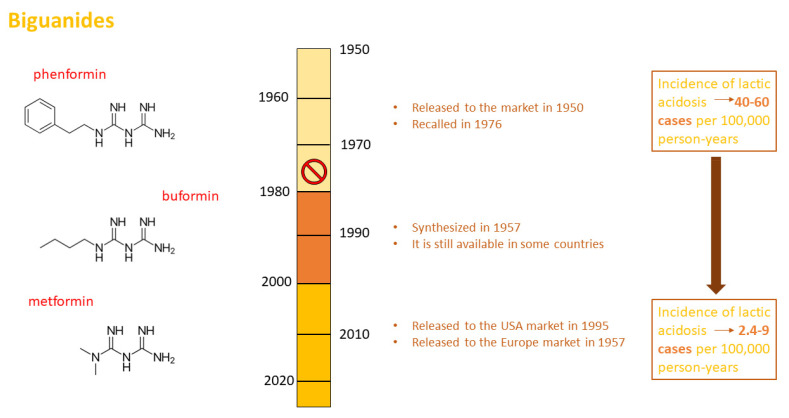
Biguanides. Chemical structures of phenformin, buformin, and metformin. Steps involved in the synthesis, release, and withdrawal from international drug markets of biguanides. Incidence of phenformin- and metformin-induced lactic acidosis per 100,000 person-years.

Commercialized for the first time in Europe in 1957, it reached true glory in 1995 when it began to be recommended and used in the United States. Moreover, buformin and phenformin belong to the same family; buformin was synthesized in 1957 but is now only commercialized in certain developing countries; thus, there is little available clinical evidence on this drug. On the contrary, phenformin was widely used during the 1950s; however, it was recalled in 1976 due to the high number of reported lactic acidosis events associated with its use (Figure 1).

**Figure 2:**
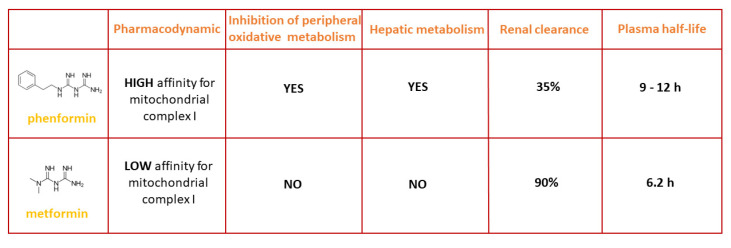
Comparison between the pharmacodynamics, inhibition of peripheral oxidative metabolism, hepatic metabolism, renal clearance, and plasma half-life of phenformin and metformin.

From a pharmacokinetic and pharmacodynamic point of view, although phenformin belongs to the same chemical class as metformin, it has a high affinity for mitochondrial complex I, with a plasmatic half-life of 9–12 h and a kidney clearance rate of only 35%; its main effect is the inhibition of peripheral oxidative metabolism, which it achieves by acting on hepatic metabolism. On the contrary, metformin has a low affinity for mitochondrial respiratory chain complex I and a plasmatic half-life of 6 h and is cleared mainly by the kidneys. Approximately 90% of metformin is eliminated within 24 h by the kidneys. Furthermore, it shows no effects on hepatic metabolism or peripheral oxidative metabolism (Figure 2). As far as buformin is concerned, there are few available data on its pharmacokinetics and pharmacodynamics [1].

Metformin has never had an equal since its first commercialization and its distribution in the American market; over the last 60 years, preclinical and clinical evidence has strongly encouraged its use [2,3,4,5,6]. In 2017, 60 years after it was first commercialized, a Special Issue of *Diabetologia* was published about it (www.springer.com/journal/125/updates/17199106 (last updated 1 January 2022)). In this review, we report on metformin’s history, its mechanisms of action, the clinical evidence for its use, and future perspectives [3,7,8,9].

## 2. Clinical Conditions for Metformin Administration and Contraindications 

The use of metformin is recommended as the first pharmacological approach after type 2 diabetes diagnosis, and the pharmacological indications and contraindications are reported on the metformin datasheet. All international diabetes guidelines agree that this approach is unique and necessary. There is also scientific evidence that suggests the use of metformin even earlier in the pre-diabetes stage [10,11]. As far as prediabetes is concerned, the recent American Diabetes Association (ADA) guidelines report that metformin therapy should be considered in adults with prediabetes for the prevention of type 2 diabetes [12]. Furthermore, in recent years, metformin has been considered for gestational diabetes treatment (GDM) and for reducing insulin resistance in pregnant women with type 2 diabetes; some international guidelines suggest the use of metformin in these conditions [13]. However, the ADA recommendations state that metformin [14] should not be used as a first-line agent because it crosses the placenta to the fetus. 

Specialists can choose to use metformin in these cases but must assume responsibility and obtain the patient’s consent.

Metformin should not be used to manage glycemia in type 1 diabetes patients, and it cannot be considered an alternative to insulin. Its administration is encouraged when a patient presents with both obesity and insulin resistance. It is widely recognized that metformin can be combined with any other diabetes drug or treatment. 

As is reported on the current product datasheets, the use of metformin is contraindicated for specific clinical conditions. Among these, we highlight the hypersensitivity to the molecule and diabetes-associated coma or pre-coma states. Furthermore, the use of metformin is not allowed in cases of chronic renal failure with an eGFR lower than 30 mL/min/1.73 m^2^, in cases of tissue hypoxia associated with diabetes (decompensated heart failure, acute respiratory failure, acute myocardial infarction or shock), or in cases of severe liver failure or alcohol intoxication. Metformin administration should generally be avoided in patients with any condition involving acute tissue hypoxia.

## 3. Interpretation of Contraindications

The contraindications for metformin use have been modified over the past few years in order to adapt them to new scientific evidence or in order to highlight incorrect clinical approaches. Concerning this latter aspect, it is important to highlight that metformin is often incorrectly considered a nephrotoxic agent that is able to damage the kidney. It is also important to highlight that this is not true. The use of metformin in recent clinical practice is not dependent on plasmatic creatinine values, and it can be used in patients with eGFR values as low as 30 mL/min/1.73 m^2^. The same concept also applies to respiratory, hepatic, and cardiac failure. In the list of contraindications, these clinical conditions are associated with the term “acute”. Hence, if we correctly interpret the contraindications paragraph in the context of the most recent datasheet, only acute respiratory, hepatic, or cardiac failure is contraindicated for metformin administration in diabetic patients.

## 4. Physiopathogenesis of “Acute” Clinical Conditions 

The contraindications for the use of metformin reported on the datasheet in different countries around the world indicate that metformin has to be administered with caution in the case of reduced kidney, liver, or pulmonary function.

However, it is widely known that these internal medical conditions are often associated with diabetes, and they can even coexist for the entire duration of a patient’s life. Thus, it is important to define the limit beyond which it is possible to administer metformin and, on the contrary, the “comfort zone” where metformin can be used for the benefit of the patient. 

In order to understand this limit, we should analyze the natural history of hepatic, pulmonary, and kidney parenchymal function failures. These organs are subjected to aging and physiological functional decreases throughout life. The functional decrease is heterogeneous and begins after the third decade of life, and the presence of systemic comorbidities and the use of complex therapies can determine a further reduction in their function. Physiologically, the limit that determines the progression from chronic to acute failure is represented by an “acute” loss of function or an “acute” increased request of function. In this condition, the higher acute request for oxygen is certainly the main cause of the rapid decline in organ function.

**Figure 3:**
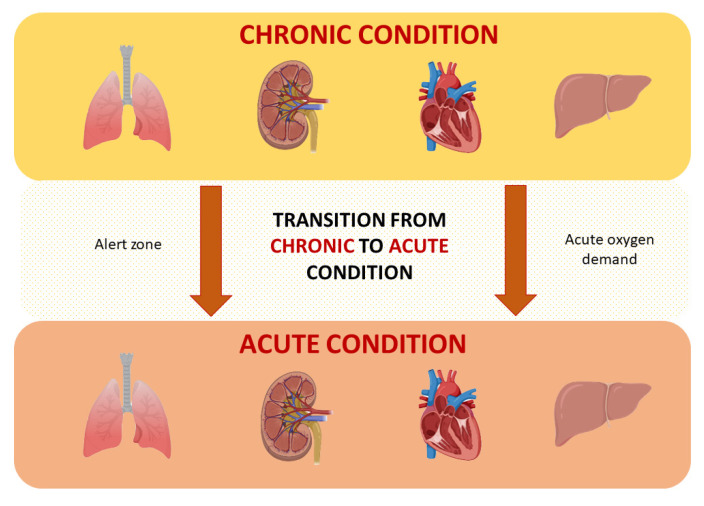
Schematic representation of the evolution from the chronic to the acute state of heart, lung, liver, and kidney damage. The transition zone represents the crucial point for the development of tissue hypoxia and acidosis.

The higher request for oxygen is commonly accepted as the main determiner of chronic to acute failure progression. This physiopathological moment at the cellular level is associated with increased lactate production. Lactate production is the main risk of biguanide use; the improper use of metformin could affect the acute oxygen needs of tissues and enhance lactate production, paving the way to lactic acidosis (Figure 3).

## 5. Lactate Production 

**Figure 4:**
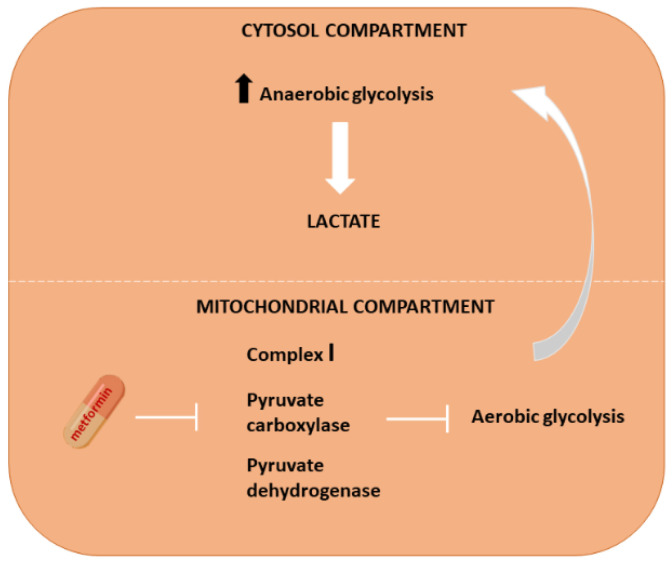
Schematic representation of intracellular lactate synthesis. Metformin inhibits the mitochondrial transport chain, inducing the blocking of aerobic glycolysis (mitochondrial compartment) and an increase in anaerobic glycolysis (the cytosolic compartment) with an accumulation of lactic acid.

As far as the cellular metabolism of nutrients is concerned, some processes occur in the cytoplasm, and others occur at the mitochondrial level. All oxidative processes occur in the mitochondria in the presence of oxygen [15]. Nutrients can be rapidly metabolized in anaerobic conditions in the cytosol. However, the most energy is obtained at the mitochondrial level through electron respiratory chain complexes. The most important reactions giving rise to this large amount of energy belong to the Krebs cycle. The main fuel of the Krebs cycle is pyruvate, which is derived from lactate produced by cytosolic anaerobic glycolysis. Pyruvate represents the first product that enters the mitochondria. In the cytosol, enhanced anaerobic metabolism is associated with increased lactate production, which has to be converted into pyruvate in order to obtain energy inside the mitochondria. The inhibition of the conversion of lactate into pyruvate could represent a limiting factor for energy production and a risk for cellular lactate accumulation. In parallel, the inhibition of pyruvate entering the mitochondria could cause an increase in pyruvate to lactate conversion and a further risk of cytosolic lactate accumulation (Figure 4). Intracellular lactate accumulation leads to pH reduction and lactic acidosis.

## 6. Lactic Acidosis 

Lactic acidosis is a clinical condition characterized by blood pH lower than 7.35 (n.v. 7.38–7.42) in the presence of arterial blood lactate levels greater than 5 mmol/L (n.v. 0.5–2.2 mmol/L; (4.5–19.8 mg/dL)) [16]. These values are obtained through arterial blood gas analysis, and they can co-present with several clinical conditions such as alveolar gas exchange deficit, kidney injury, dehydration (vomiting and diarrhea), and sepsis [17]. Therefore, restoration of effective circulating volume and hydration are the solutions to many of these conditions. In hypoxic conditions, the reoxygenation of the hypoxic tissue seems to be the correct intervention strategy. Regarding the acute kidney injury induced by contrast media in radiology, the use of exogenous bicarbonate and appropriate hydration could represent the correct approach in these patients.

Lactic acidosis is a medical emergency that can lead to patient death, and the presence of metformin or biguanides in the bloodstream could worsen patient outcomes. Therefore, an in-depth knowledge of the role of metformin in lactic acidosis is necessary for correct patient management.

## 7. Lactic Acidosis and Metformin 

**Figure 5:**
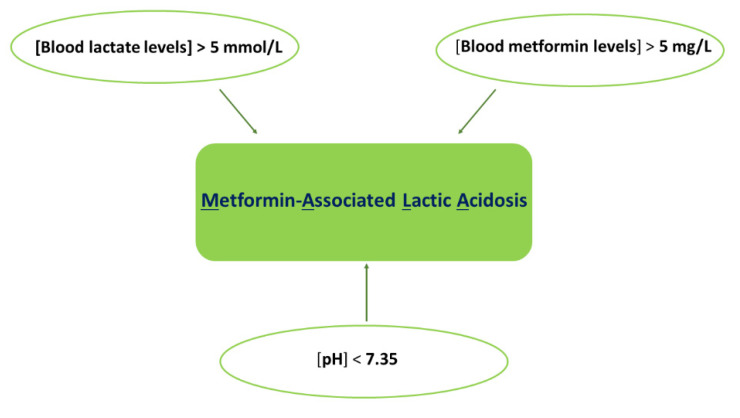
Lactic acidosis is associated with the use of metformin (MALA). MALA is determined by blood lactate levels > 5 mmol/L, pH < 7.35, and blood metformin levels > 5 mg/L.

The simultaneous presence of metformin and lactic acidosis is defined as metformin-associated lactic acidosis (MALA) [17]. In this condition, a cut-off value for blood metformin levels should be established in addition to blood pH and lactate levels (Figure 5).

This value is not well defined in clinical practice because previous studies have not been able to indicate a specific value of metformin in the blood. There are several variabilities in the analysis of patients with MALA, such as dosage and determination of metformin. Moreover, it is also unclear whether it is better to use metformin measurements in plasma or serum. Lalau et al. reported heterogeneity of the analyzed values of metformin. More specifically, the plasma concentrations and erythrocyte levels of metformin were 2.7 and 2.0 mg/L, respectively. Notwithstanding this variability, the study concluded that values of metformin in the blood higher than 5 mg/L indicate a marked risk of MALA [18].

## 8. MALA in the Real World

As has already been reported, phenformin treatment may induce lactic acidosis (40–60 cases/100,000 patients/year) [19,20], while lactic acidosis events under metformin therapy are much rarer in developed countries (2.4–9 cases/100,000 patients/year) [21,22,23]. Nevertheless, metformin use is associated with a low risk of lactic acidosis. When lactic acidosis occurs, it is always a high-risk acute condition that can determine death. For this reason, it is necessary to know about MALA in depth. Therefore, considering the triad (pH < 7.35, blood lactate levels > 5 mmol/L, blood metformin levels > 5 mg/L) that determines MALA, it is important to better understand whether these values are dependent on or independent of metformin treatment. Huang et al. examined 2747 articles to determine whether metformin treatment was associated with lactic acidosis [24]. In particular, the authors analyzed lactate levels in patients treated chronically with metformin, dividing studies into the following groups: (1) patients who did not have contraindications to metformin treatment, (2) patients who had contraindications to metformin treatment, (3) patients undergoing exercise influencing lactate production, and (4) HIV-positive patients and those receiving nucleoside reverse transcriptase, which is able to inhibit mitochondrial metabolism. In all patient groups, examined lactate levels never exceeded the threshold risk value of 5 mmol/L, indicating that metformin is associated with a reduced risk of producing high levels of lactate and consequent MALA.

## 9. What Happens to the Triad during MALA? 

It is important to analyze the triad data in more detail in the course of MALA. Duong et al. described a cohort of patients previously hospitalized for MALA who satisfied the triad (mean plasma concentration of metformin = 29.8 ± 19.1 mg/L, mean lactate concentration = 12.9 ± 6.1 mmol/L, mean pH = 7 ± 0.2) [17]. In all analyzed patients, the metformin, lactate, and pH values were serially determined in association with functional, disease progression, and clinical prognosis data. In this cohort, patient severity was not significantly correlated with pH, lactate concentration, or metformin levels. Moreover, the metformin plasma concentration was not positively related to the lactate levels; however, it was negatively correlated with pH. The main determinant of poor prognosis was kidney functional decline. AKI (acute kidney injury), and vomiting and diarrhea were associated with a worse prognosis. Therefore, as was highlighted at the beginning of this article, perhaps it is the decline in the acute phase of a clinical condition that represents the real determinant of the risk of the MALA triad.

## 10. Metformin, Sulfonylureas, and Lactic Acidosis

As is reported above, patients treated with phenformin showed a higher risk of developing MALA when compared with those treated with metformin. However, it is important to report the risk of lactic acidosis associated with antidiabetic therapies other than the biguanide family. A recent study compared the risk of lactic acidosis-associated hospitalization in patients under metformin treatment and patients under sulfonylureas treatment, evaluating the development of reduced kidney function [25]. In this research, data from a retrospective cohort of about 50,000 American veterans were examined through propensity score matching. A total of 24,542 new users of metformin and 24,662 users of sulfonylureas were analyzed. The endpoints of this study were lactic acidosis hospitalization or lactic acidosis development and death. In conclusion, among veterans with diabetes and reduced kidney function, the events of lactic acidosis hospitalization were not significantly different between patients treated with metformin or sulfonylureas. 

## 11. Metformin: Can We Stay Safe? 

For all the above reasons, the risk of developing MALA in patients treated with metformin appears to be low. Kidney function and the pathological conditions that can lead to acute kidney injury seem to be the only conditions that have to be evaluated during metformin therapy. Therefore, in subjects with normal glomerular filtrate and hydration, metformin can be safely used at the correct dosage. Instead, in subjects with reduced glomerular filtrate (eGFR < 30 mL/min/1.73 m^2^), with the risk of developing acute kidney injury, sepsis, or other conditions that determine plasma metformin accumulation, it may be necessary to perform a lactate concentration test and arterial blood gas analysis. Accordingly, treatment with metformin is not correlated with a risk of lactic acidosis or kidney injury onset.

In light of the above reported data, we can affirm that it is not correct to directly associate metformin use with lactic acidosis and kidney injury risk. The data reported in this review clarify the effective role of metformin in the context of lactic acidosis. These observations have also been elegantly described by Lalau et al. [18]. The authors of this article conclude that metformin can be considered safe when correctly used, and they suggest the reclassification of lactic acidosis cases not correlated with the use of metformin but observed in patients that use this drug. In these cases, the authors suggest using the acronym MULA (metformin-unrelated lactic acidosis) instead of MALA, highlighting that metformin use has no causative role in acidosis pathogenesis. For uncertain cases where it is impossible to exclude the involvement of metformin (i.e., in case of sepsis, hemodynamic shock or in general hypossiemic conditions), it would be appropriate to use the acronym MILA (metformin-induced lactic acidosis). However, as has been reported in this review, these cases are rare.

In conclusion, it may be necessary to discriminate between MALA, MULA, and MILA in clinical practice in order to evaluate the risk associated with metformin treatment.

## 12. Conclusions

After more than 60 years, metformin remains one of the most important drugs in clinical practice. Fifty years of research has highlighted the beneficial effects of metformin treatment in diabetic patients.

For this reason, metformin represents a striking example of a “historical nemesis” of a drug. About 40 years after its marketing in Europe, once its efficacy and safety had been demonstrated, metformin was registered in the US as an anti-diabetes drug. Recently, several reviews have collected the most up-to-date scientific evidence in favor of the action of metformin as an endothelial protector [26], as an effective drug in heart failure, as an anti-inflammatory useful in rheumatological/immunological diseases, and in general as a beneficial drug against many aging-related morbidities [27].

Moreover, metformin is frequently used in other pathologies. For this reason, metformin treatment will be expanded in the future to manage many diseases. Nevertheless, the risk of lactic acidosis is always an important parameter to consider. Current evidence supports the safe treatment of even weak patients with metformin. Moreover, it is important to take into account the possibility of suspending metformin treatment in cases involving clinical conditions that could require an increased oxygen consumption or progression from equilibrium to acute status. Excluding these aspects, it is possible to allow the use of metformin treatment in all patients with the correct indications.

## Data Availability

Not applicable.
